# ﻿*Ligularialushuiensis* (Asteraceae, Senecioneae), a new species from northwestern Yunnan, China

**DOI:** 10.3897/phytokeys.238.117340

**Published:** 2024-02-07

**Authors:** Xiao-rui Chi, Hai-song Wu, Long Wang

**Affiliations:** 1 Key Laboratory of Plant Resources Conservation and Sustainable Utilization, South China Botanical Garden, Chinese Academy of Sciences, Guangzhou 510650, Guangdong, China South China Botanical Garden, Chinese Academy of Sciences Guangzhou China; 2 University of Chinese Academy of Sciences, Beijing 100049, China University of Chinese Academy of Sciences Beijing China

**Keywords:** Compositae, Sino-Himalayan flora, taxonomy, Yunnan

## Abstract

*Ligularialushuiensis*, a new species from northwestern Yunnan, China, is described and illustrated. It was tentatively placed in L.sect.Ligulariaser.Ligularia on the basis of the pinnate-palmate leaf venation, racemose synflorescence and pappus which is as long as tubular corolla. Within the series, it appeared somewhat close to both *L.lamarum* and *L.pseudolamarum*. However, *L.lushuiensis* can be easily distinguished from the latter two species by, among other characters, the leaf margin, bract size, involucre shape and size, and number and width of ray florets. Morphologically, *L.lushuiensis* is also superficially similar to *L.secunda* but differs readily by having distally shortly yellowish and brownish puberulent stems, palmately-pinnately veined leaves regularly dentate at margin, scarious, brown and larger bracts, and larger ray laminae. In addition, a distribution map and a diagnostic key to Chinese species of L.ser.Ligularia are also provided.

## ﻿Introduction

*Ligularia* Cass. (Asteraceae, Senecioneae), with approximately 130 species recognized, is mainly distributed in eastern Asia (Liu 1989; Liu et al. 1994; Liu and Illarionova 2011; Ren et al. 2020). The center of species diversity of the genus lies in the eastern Himalayas and the Hengduan Mountains region in southwestern China (Liu et al. 1994, 2006; Liu and Illarionova 2011). In the last decade, many taxonomic revisions at specific level have been continuously carried out in the genus (see Fei et al. 2019; Lazkov and Sennikov 2019; Guo and Wang 2022; and literature cited therein).

**Figure 1.**
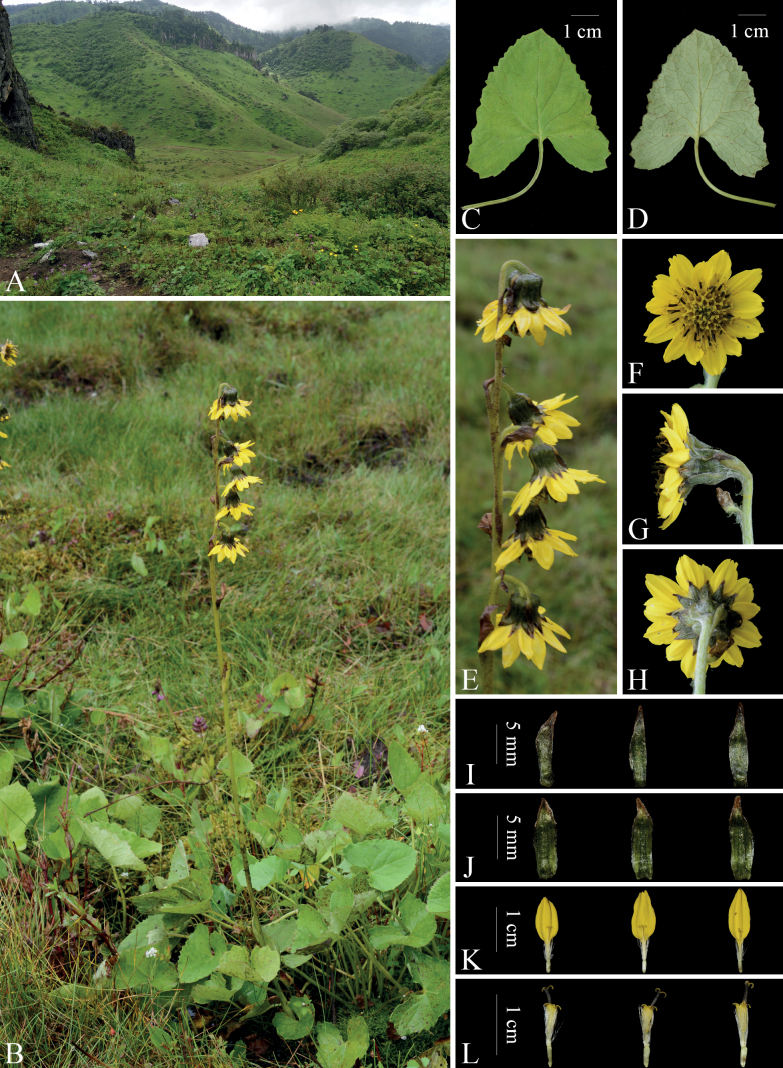
*Ligularialushuiensis* sp. nov. **A** habitat **B** habit **C** basal leaf (adaxial surface) **D** basal leaf (abaxial surface) **E** synflorescence **F** capitulum (top view) **G** capitulum (side view) **H** capitulum (back view) **I** outer phyllaries (abaxial surface) **J** inner phyllaries (abaxial surface) **K** ray florets **L** tubular florets. Photographs by Long Wang.

During a botanical expedition to northwestern Yunnan, China in 2017, we discovered an unusual population of *Ligularia* in a less-botanized area in Lushui city (Fig. 1). It appeared to be similar to both *L.pseudolamarum* Long Wang & X.Q.Guo and *L.secunda* Y.S.Chen in the general habit, especially in the capitula that are oriented to one side of the synflorescence axis. However, they showed great differences in an array of characters. The plants also displayed a slight resemblance to *L.lamarum* (Diels) C.C.Chang, but can also be easily distinguished from that species. We therefore determine that the plants in question represent a hitherto undescribed species, which we describe below.

## ﻿Material and methods

For morphological comparison, we critically examined physical or digitalized herbarium specimens deposited at several major herbaria in China, including CDBI, HNWP, IBSC, KUN, NAS, PE, SZ, and WUK (acronyms follow Thiers (2023)). Specimens of *L.lushuiensis* were collected and photographed during our field investigation to Yunnan province in 2017. Morphological observations and measurements were based on fresh material as well as herbarium specimens deposited at IBSC.

## ﻿Taxonomic treatment

### ﻿ Ligularia lushuiensis

Taxon classificationPlantaeAsteralesAsteraceae

Long Wang sp. nov.

F8BB2F03-9DD7-5A8E-8D2D-F88F7BDE98E3

urn:lsid:ipni.org:names:77335783-1

Figs 1 , 2

#### Diagnosis.

*Ligularialushuiensis* should be placed within L.ser.Ligularia owing to character combination of palmate-pinnate leaf venation, scarious and brown bracts, single-oriented capitula, and broadly cylindrical involucres 1–1.1 cm high and 1.1–2 cm in diam. Morphologically, it is somewhat similar to *L.lamarum*, *L.pseudolamarum*, and *L.secunda*. From *L.lamarum*, it differs in the leaf margin, bract texture, color, and size, involucre shape and size, and ray floret number and width; from *L.pseudolamarum*, it differs in the leaf shape and margin, bract size, involucre shape and size, and ray floret number and size; and from *L.secunda*, it differs in the stem indumentum, leaf venation and margin, bract texture, color and size, and ray floret size.

#### Type.

**Figure 2.**
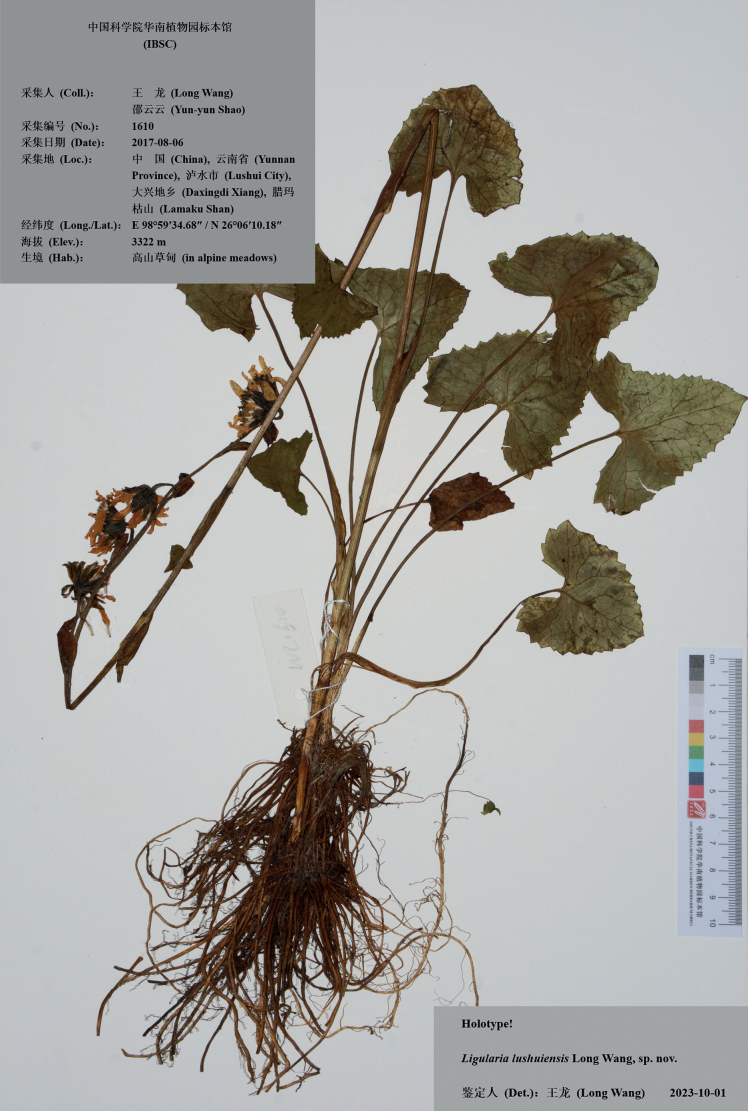
Holotype sheet of *Ligularialushuiensis* sp. nov.

China. Yunnan: Lushui, Daxingdi, Lamaku Shan, 26°06'10.18"N, 98°59'34.68"E, alpine meadows, 3322 m a.s.l., 6 August 2017 (fl.), *Long Wang & Yun-yun Shao 1610* (holotype: IBSC; isotypes: IBSC). Fig. 2.

#### Description.

Perennial herb. Stems solitary or 2, erect, 30–70 cm tall, 4–5 mm in diam. at base, proximal to middle part yellowish puberulent, distal part shortly yellowish and brownish puberulent. Basal leaves petiolate; petiole 5–12 cm long, not winged throughout; leaf blade ovate-cordate, 5–8(–10) cm long, 4.5–8(–11) cm wide, herbaceous, adaxially dark green, glabrous, abaxially greenish, slightly brownish puberulent only on veins, palmately-pinnately veined, base cordate, margin regularly dentate, apex obtuse; sinus narrow, basal lobes suborbicular, divergent. Stem leaves 3–6. Proximal stem leaves 1–2, similar to but smaller than basal leaves. Median stem leaves 1–2, shortly petiolate or sessile, base tubular-amplexicaul. Distal stem leaves 1–2, bracteal, scarious. Capitula (2–)5–9, in a lax raceme, oriented to one side of the synflorescence axis; peduncles short, ca. 1 cm long; bract 1, ovate-lanceolate, ca. 1 cm long, 6–7 mm wide, scarious, brown; bracteoles 2 or 3, oblong-lanceolate, ca. 1.1 cm long, 3 mm wide, scarious, brown. Involucres broadly cylindrical, 1–1.1 cm high, 1.1–2 cm in diam., outside more or less whitish arachnoid; receptacle whitish arachnoid outside; phyllaries 12–15, in 2 rows; outer phyllaries narrowly oblong, 2–2.5 mm wide, apex acute; inner phyllaries oblong, ca. 3 mm wide, margin membranous, apex acute to obtuse. Ray florets 10–13, yellow; lamina ovate-oblong, 1.5–1.6 cm long, 5–6 mm wide, apex obtuse, 2- or 3-denticulate; tube ca. 4 mm long. Tubular florets numerous, yellow, ca. 1 cm long; tube 2–3 mm long; limb campanulate, 4–5 mm long; style 6–7 mm long, branches dark yellow. Achenes (immature) narrowly oblong, 3.5–4 mm long, glabrous. Pappi white, ca. 7 mm long, as long as or slightly shorter than tubular corolla.

#### Distribution and habitat.

**Figure 3.**
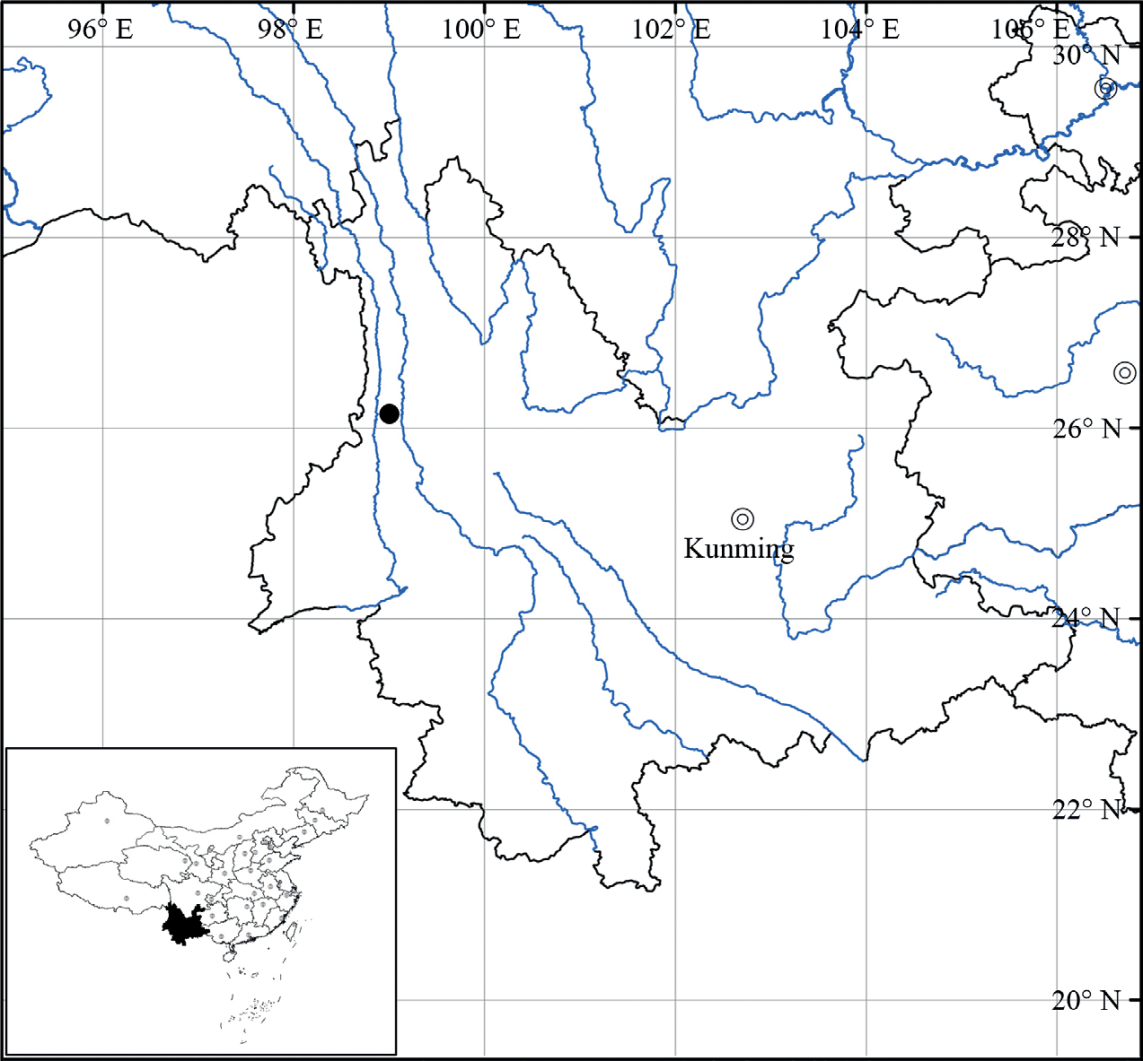
Distribution of *Ligularialushuiensis* sp. nov. (black dot).

*Ligularialushuiensis* is currently known only from its type locality, i.e. Lushui, northwestern Yunnan, China (Fig. 3). It grows in alpine meadows at an elevation of ~3322 m above sea level.

#### Etymology.

The specific epithet ‘*lushuiensis*’ refers to the type locality of this new species, i.e. Lushui City.

#### Phenology.

Flowering from July to August; fruiting from late August to September.

#### Vernacular name.

泸水橐吾 (Chinese pinyin: lú shuǐ tuó wú).

#### Conservation status.

*Ligularialushuiensis* is currently known only from a small population at its type locality, i.e. Lamaku Shan. The single population we discovered consists of no more than 200 mature individuals. Overgrazing might be the major threat to the habitat of this species. According to the IUCN Red List Categories and Criteria (IUCN 2019), this species should be categorized as Critically Endangered (CR): B1ab(iii)+2ab(iii).

#### Notes.

Morphologically, *Ligularialushuiensis* resembles both *L.pseudolamarum* and *L.secunda*, especially in the single-oriented capitula and racemose synflorescences. It is also superficially similar to *L.lamarum*, especially in the general habit and in the leaf shape and synflorescence type. Table 1 provides detailed morphological comparisons among these four species.

**Table 1.** Morphological differences among *Ligularialamarum*, *L.lushuiensis* sp. nov., *L.pseudolamarum*, and *L.secunda*.

	*L.lamarum*	*L.lushuiensis*	*L.pseudolamarum*	*L.secunda*
Stems	distally white arachnoid or brown puberulent, 2–4 mm in diam. at base	distally shortly yellowish and brownish puberulent, 4–5 mm in diam. at base	distally shortly yellowish and brownish puberulent, 5–6 mm in diam. at base	distally shortly and sparsely white arachnoid-puberulent, 5–6 mm in diam. at base
Basal leaves	triangular-sagittate or ovate-cordate, 3–9 cm long, 2.2–12.5 cm wide, adaxially and abaxially glabrous, palmately veined, base cordate, margin regularly denticulate, apex acute	ovate-cordate, 5–8 (–10) cm long, 4.5–8 (–11) cm wide, adaxially glabrous, abaxially slightly brownish puberulent only on veins, palmately-pinnately veined, base cordate, margin regularly dentate, apex obtuse	triangular-sagittate, 3–8 cm long, 3–8 (–10) cm wide, adaxially slightly whitish puberulent to glabrescent, abaxially slightly whitish puberulent to glabrescent, palmately-pinnately veined, base cordate, margin coarsely dentate, apex sharply acute	ovate, 4.5–10 cm long, 3–7 cm wide, adaxially shortly puberulent, abaxially glabrous, pinnately veined, base truncate or shallowly cordate, margin denticulate, apex acute
Stem leaves	petiolar base tubular-amplexicaul	petiolar base tubular-amplexicaul	petiolar base tubular-amplexicaul	petiolar base semi-amplexicaul
Synflorescence	usually many-flowered, oriented to one side of the synflorescence axis	(2–) 5–9, oriented to one side of the synflorescence axis	(1–) 2–6 (–10)-flowered, oriented to one side of the synflorescence axis	5–10-flowered, turning to one side of the synflorescence axis
Bracts	subulate, leaflike, green, 1–1.5 cm long, 1–2 mm wide	ovate-lanceolate, scarious, brown, ca. 1 cm long, 6–7 mm wide	ovate-lanceolate, scarious, brown, ca. 3 cm long, 6–7 mm wide	boat-shaped to linear, leaflike, green, 2.5–4.5 cm long, ca. 1 cm wide
Involucres	campanulate-turbinate, 6–9 mm high, 3–5 mm in diam., outside glabrous	broadly cylindrical, 1–1.1 cm high, 1.1–2 cm in diam., outside more or less whitish arachnoid	narrowly cylindrical, 9–11 mm high, 3–4 mm in diam., outside slightly shortly yellowish puberulent to glabrescent	broadly cylindrical, 1.2–1.5 cm high, 1.5–2 cm in diameter, outside sparsely arachnoid-puberulent
Ray florets	5–8; lamina 7–10 mm long, ca. 1.5 mm wide	10–13; lamina ovate-oblong, 1–1.2 cm long, 5–6 mm wide	3–5; lamina oblong to elliptic, 7–8 mm long, 2.5–3 mm wide	8–9; lamina oblong, 1.1–1.3 cm long, 3–4 mm wide
Pappus	yellowish or brownish, 6–7 mm long	white, ca. 7 mm long	white, 7–8 mm long	white, 8 mm long

In the genus *Ligularia*, *L.confertiflora* C.C.Chang is also recorded to have capitula that are oriented to one side of the synflorescence axis except for *L.lushuiensis*, *L.pseudolamarum*, and *L.secunda*. However, this species is characterized by having palmate leaf venation and short pappus which is as long as the tube of tubular corolla and is readily placed in L.ser.Speciosae Pojark. It is easily distinguishable from *L.lushuiensis* in having discoid capitula and leaflike bracts.

According to the infrageneric classification proposed by Liu (1985), *Ligularialushuiensis* is tentatively assigned to L.sect.Ligulariaser.Ligularia because of the character combination of pinnate-palmate leaf venation, racemose synflorescence and pappus which is as long as tubular corolla. It is noteworthy that the pinnate-palmate leaf venation appears frequently in several species within this series. With the addition of this new species, 14 species are currently recognized in the series in China (Liu 1988; Grierson and Springate 2000; Guo and Wang 2022). We herein provide a diagnostic key to the Chinese species of L.ser.Ligularia to facilitate identification of this group of plants.

### ﻿Key to Chinese species of L.ser.Ligularia

**Table:** 

1	Bracts ovate, ovate-oblong to ovate-lanceolate, 6–10 (–20) mm wide	**2**
–	Bracts linear-lanceolate to linear, usually less than 5 mm wide	**5**
2	Leaf blades triangular-sagittate; basal lobes of sinuses sagittate; involucres narrowly cylindrical, 3–4 mm in diam., outside slightly shortly yellowish puberulent to glabrescent	***L.pseudolamarum***
–	Leaf blades ovate-cordate, triangular-cordate, reniform-cordate, broadly cordate, or reniform; basal lobes of sinuses oblong or suborbicular; involucre broadly cylindrical, campanulate, campanulate-turbinate, or cupular, 6–20 mm in diam., outside glabrous, more or less whitish arachnoid, or sparsely shortly puberulent	**3**
3	Leaves palmately-pinnately veined; capitula (2–) 5–9, oriented to one side of the synflorescence axis	***L.lushuiensis***
–	Leaves pinnately veined; capitula numerous, not specifically oriented	**4**
4	Leaf blades abaxially glabrous; bracts herbaceous, green; involucres broadly campanulate, campanulate, campanulate-turbinate, outside glabrous	***L.sibirica***
–	Leaf blades abaxially slightly yellow pilose; bracts membranous, purplish red; involucres cupular, outside sparsely shortly pilose	***L.cyathiceps***
5	Capitula in racemes or solitary, with ray florets, rarely without florets (in *L.subspicata*)	**6**
–	Capitula usually in paniculate racemes, without ray florets	**13**
6	Leaf blades ovate-cordate, triangular-cordate to triangular, hastate or sagittate, apically usually acute or obtuse, rarely rounded	**7**
–	Leaf blades reniform or cordate-reniform, apically usually rounded	**9**
7	Leaf bases truncate, rarely cuneate or cordate; pappi purplish red	***L.parvifolia***
–	Leaf bases cordate; pappi usually whitish or yellowish	**8**
8	Ray florets present	***L.lamarum***
–	Ray florets absent, or limbs of outer tubular florets divided, labiate	***L.subspicata***
9	Stems robust, to 1 cm in diam. at base; involucres broadly campanulate to turbinate	***L.wilsoniana***
–	Stems slender, 1.5–4 (–6) mm in diam. at base; involucres usually campanulate to narrowly campanulate, rarely hemispheric (in *L.latiligulatum*)	**10**
10	Ray laminae short, small, inconspicuous	***L.atkinsonii***
–	Ray laminae normal, conspicuous	**11**
11	Abaxial surfaces of leaves densely shortly white pilose	***L.pubifolia***
–	Abaxial surfaces of leaves glabrous or slightly shortly pilose between teeth of leaf margins	**12**
12	Involucres narrowly cylindrical; ray laminae linear, apically acuminate	***L.hookeri***
–	Involucres hemispheric; ray laminae broadly oblanceolate, apically truncate	***L.latiligulata***
13	Distal stems, synflorescences and abaxial sides of involucres shortly brown pilose; leaves 5–11 cm wide, adaxially glabrous; pappus yellow	***L.leveillei***
–	Distal stems and synflorescences densely yellow pilose, and involucres glabrous; leaves ca. 5 cm wide, adaxially shortly yellow pilose; pappus brown	***L.nanchuanica***

## Supplementary Material

XML Treatment for Ligularia lushuiensis

## ﻿Additional information

### Author ORCIDs

Xiao-rui Chi https://orcid.org/0000-0002-8013-4484

Hai-song Wu https://orcid.org/0000-0003-3023-7220

Long Wang https://orcid.org/0000-0001-6059-0020

### Data availability

All of the data that support the findings of this study are available in the main text.
